# Exploring Biological Impacts of Prenatal Nutrition and Selection for Residual Feed Intake on Beef Cattle Using Omics Technologies: A Review

**DOI:** 10.3389/fgene.2021.720268

**Published:** 2021-11-01

**Authors:** Aidin Foroutan, David S. Wishart, Carolyn Fitzsimmons

**Affiliations:** ^1^ Department of Pathology and Laboratory Medicine, Western University, London, ON, Canada; ^2^ Department of Biological Sciences, University of Alberta, Edmonton, AB, Canada; ^3^ Department of Computing Science, University of Alberta, Edmonton, AB, Canada; ^4^ Department of Agricultural Food and Nutritional Science, University of Alberta, Edmonton, AB, Canada; ^5^ Agriculture and Agri-Food Canada, Edmonton, AB, Canada

**Keywords:** feed efficiency, RFI, prenatal nutrition, beef cattle, omics

## Abstract

Approximately 70% of the cost of beef production is impacted by dietary intake. Maximizing production efficiency of beef cattle requires not only genetic selection to maximize feed efficiency (i.e., residual feed intake (RFI)), but also adequate nutrition throughout all stages of growth and development to maximize efficiency of growth and reproductive capacity, even during gestation. RFI as a measure of feed efficiency in cattle has been recently accepted and used in the beef industry, but the effect of selection for RFI upon the dynamics of gestation has not been extensively studied, especially in the context of fluctuating energy supply to the dam and fetus. Nutrient restriction during gestation has been shown to negatively affect postnatal growth and development as well as fertility of beef cattle offspring. This, when combined with the genetic potential for RFI, may significantly affect energy partitioning in the offspring and subsequently important performance traits. In this review, we discuss: 1) the importance of RFI as a measure of feed efficiency and how it can affect other economic traits in beef cattle; 2) the influence of prenatal nutrition on physiological phenotypes in calves; 3) the benefits of investigating the interaction of genetic selection for RFI and prenatal nutrition; 4) how metabolomics, transcriptomics, and epigenomics have been employed to investigate the underlying biology associated with prenatal nutrition, RFI, or their interactions in beef cattle; and 5) how the integration of omics information is adding a level of deeper understanding of the genetic architecture of phenotypic traits in cattle.

## Introduction

Feed is, by far, the largest financial burden for the beef industry (Canfax Research Services, 2017; Ozeran et al., 2019; OMAFRA, 2021). The cost of feed varies from region-to-region with dependencies on climate and food source availability. It also varies from year-to-year, with a strong connection to food resource unit costs (Canfax Research Services, 2017; Ozeran et al., 2019; OMAFRA, 2021). In particular, a longer winter-feeding period increases feed costs. To lower winter feed costs and improve profitability, some producers are using extended grazing seasons as well as utilizing alternative feeds (Canfax Research Services, 2017; OMAFRA, 2021).

Cattle producers need to find the balance between meeting a herd’s feed or nutritional needs while doing so in a cost-effective manner. Analyzing the chemical composition of feed and forage to understand their nutrient content is another strategy for more optimal cow/calf feed management (Stanton and LeValley, 2014; Heeg, 2016; Canfax Research Services, 2017). This approach allows producers efficiently meet the nutrient requirement of their animals and decrease feed expenses. Therefore, proper management of feed costs is crucial to improve profitability in the beef industry.

In addition to adopting optimized feed management strategies to reduce feed costs and increase profitability, genetic selection (for animals with optimal feeding responses) has also been used to improve farm profitability. In particular, the selection for animals which eat less but produce the same amount of meat has attracted considerable attention from beef producers. One such measure of feed conversion efficiency is called residual feed intake (RFI). The use of RFI in livestock breeding programs is growing rapidly, especially in the beef industry, since those cattle which are genetically classified as Low-RFI (LRFI, efficient animals) eat less and produce less methane per unit weight gain (Basarab et al., 2013). However, because RFI selection is relatively new to the beef industry it will be important to investigate if other traits, either positive or negative, may be co-selected along with LRFI. A concerning issue is the effect of selection for RFI on the physiological status of the cow during gestation, and how that might be impacted by available feed intake and/or nutritional status of the animal. As well, although both RFI and maternal nutrition during gestation may affect progeny traits, our understanding of the biological mechanisms by which this occurs is poor.

As noted earlier, the highest feed cost period typically occurs during winter when animals cannot eat fresh forage. In colder regions of the world such as Canada, northern regions of the United States and some European countries, winter usually coincides with the second half of a cow’s gestation period when the most critical nutrient needs of dams should be provided. Almost 75% of ruminant fetal growth occurs in late gestation, and therefore nutrient insults at this stage will have the greatest impact on fetal size (i.e., birth weight and body length) (Du et al., 2010; Funston et al., 2010). In contrast, modest malnutrition or nutrition restriction during first half of gestation is of little significance postnatally since the fetus needs relatively little nutrition for growth and development at this stage. Indeed, studies have shown that calves born from dams that were nutritionally restricted during early-to mid-gestation then re-alimented before parturition had similar ratios of weight gain to feed intake compared to fully-fed control groups (Long et al., 2009; Long et al., 2012). However, it is not clear how lower planes of nutrition during early gestation alone or in combination with genetic selection for LRFI could potentially affect key efficiency and production traits of the progeny animals and this possibility needs additional research.

An improved understanding of the physiological basis of RFI, prenatal nutrition, and their effect upon progeny traits can be achieved through the help of multiple omics sciences including metabolomics, transcriptomics and epigenomics. This review focuses on: 1) the importance of RFI as a measure of feed efficiency and how it can affect other economic traits in beef cattle; 2) the influence of prenatal nutrition on physiological phenotypes in calves; 3) the benefits of investigating the interaction of genetic selection for RFI and prenatal nutrition; 4) how metabolomics, transcriptomics, and epigenomics have been employed to investigate the underlying biology associated with prenatal nutrition, RFI, or their interactions in beef cattle; and 5) how the integration of omics information is adding a level of deeper understanding of the genetic architecture of phenotypic traits in cattle.

### RFI as a Measure of Feed Efficiency

RFI is a feed efficiency measure calculated using the difference between an animal’s actual feed intake and its expected feed requirements for maintenance and growth over a specific time period (Koch et al., 1963; Arthur et al., 1996; Basarab et al., 2003; Nkrumah et al., 2006). RFI is independent of growth characteristics such as body weight (BW) and ADG (Koch et al., 1963; Kennedy et al., 1993; Crews, 2005). Indeed, a study by Herd et al. (2014) showed that selection for LRFI in beef cattle reduced feed intake without compromising body size or growth. Other studies have shown no differences in growth, carcass yield and quality grade between LRFI and HRFI cattle (Basarab et al., 2003; Nkrumah et al., 2007). In a study conducted by Castro Bulle et al. (2007), similar dressed carcass yield, backfat thickness, yield grades, and marbling scores were seen between LRFI and HRFI cattle. As well, no differences could be detected in muscle depth and fat depth as well as in ultrasonic fat measures between LRFI and HRFI cattle (Fitzsimons et al., 2013). However, a positive correlation between ultrasound backfat measures and RFI was reported by Basarab et al. (2003) and Arthur et al. (2001). Richardson et al. (2001) also reported that Angus steers born from LRFI parents had more whole-body protein and less whole-body fat compared to progeny steers of HRFI parents. These conflicting results suggest that further research is needed to understand the relationship between body fat and RFI.

RFI has a moderate heritability (h^2^ = 0.29–0.46) in cattle, which makes it a good candidate for genetic improvement (Arthur et al., 2001; Basarab et al., 2003; Nkrumah et al., 2007). Selecting for LRFI (more efficient) animals is gaining popularity among beef producers and is expected to increase (Herd and Arthur, 2009; Basarab et al., 2013), since those cattle which are genetically classified as LRFI eat less and produce less methane per unit weight gain (Basarab et al., 2013). Differences in RFI appear to be due to differences in energy partitioning, basal metabolism, body composition, as well as activity and energy expenditure (Basarab et al., 2003; Herd et al., 2004; Kelly et al., 2011).

#### Physiological Basis for RFI

The differences in energy requirement for divergent RFI animals are related to physiological processes including intake of feed, activity, digestion of feed, body composition and metabolism, and thermoregulation. Although these were reviewed in depth by Herd and Arthur (2009) as well as Alende et al. (2016), here we report a summary of key findings about the association between RFI and physiological processes. In terms of feeding activity, high-RFI (HRFI) cattle have a higher energy requirement (2–5% more) as compared to their LRFI counterparts which is in part due to the higher frequency of daily feeding events (events/day), and the longer duration of head-down time (min/day) (Herd et al., 2004; Nkrumah et al., 2007; Kelly et al., 2010; Basarab et al., 2011; Manafiazar et al., 2015). For the general activity of animals, a positive correlation between RFI and daily pedometer count has been reported in cattle (Richardson et al., 2000). Several studies investigating the digestion of feed have shown a negative correlation between RFI and digestibility in cattle, indicating that LRFI cattle tend to have greater feed digestibility (Nkrumah et al., 2006; Johnson et al., 2019; McDonald et al., 2010; Basarab et al., 2013). With reference to body composition and metabolism, LRFI heifers also have less intramuscular and subcutaneous fat, as well as lower feeding frequency than HRFI heifers (Basarab et al., 2011). Finally yet importantly regarding thermoregulation, DiGiacomo et al. (2014) reported that HRFI Holstein-Friesian cows had higher skin, neck, and shoulder temperatures as compared to LRFI cows. Although there might be few conflicting reports regarding energy requirements and expenditures for divergent RFI cattle, most studies have reported less energy requirements and expenditures in LRFI cattle.

#### The Relationship Between RFI and Enteric Methane

As the relationships between RFI and enteric methane have not been extensively reviewed, we will provide a deeper description as compared to the physiological basis of RFI. Methane (CH_4_) is an important greenhouse gas that contributes to global warming. The global warming potential of methane is about 25-fold higher than that of CO_2_ (Intergovernmental Panel on Climate Change, 2014). Livestock are responsible for approximately 14–22% of greenhouse gas emissions (Shafer et al., 2011; Knapp et al., 2014). The majority of greenhouse gas emissions from cattle come from enteric methane, which is produced in ruminants by rumen microbes during digestion and fermentation of their food (Beauchemin et al., 2010). Therefore, apart from reducing feed costs, selecting for LRFI cattle can also reduce methane production. Nkrumah et al. (2006), Basarab et al. (2013), and Dini et al. (2019), all reported that LRFI cattle had 15–28% lower methane production compared to HRFI cattle in various beef production scenarios. The lower level of methane production in LRFI cattle is most likely due to the fact that LRFI animals have lower dietary energy intake at equal levels of production compared to HRFI animals (Herd et al., 2002; Intergovernmental Panel on Climate Change, 2014). Manafiazar et al. (2020) reported lower methane and CO_2_ emissions from LRFI yearling beef heifers and mature cows compared to their HRFI counterparts, which, for a large part, was due to lower feed consumption at equal body weight, gain, and fatness in LRFI animals. Manafiazar et al. (2021) also reported that LRFI pregnant heifers consumed less forage (10.25 vs. 10.81 kg DM d −1; *p* < 0.001), and emitted less daily methane (238.7 vs. 250.7 g d −1; *p* = 0.009) and CO_2_ (7,578 vs. 8,041 g d −1; *p* < 0.001) compared to HRFI animals. Lower feed intake results in a host-mediated response in microbial communities (bacteria, ciliate protozoa, fungi, and archaea) in LRFI animals favoring reduced methane production (Russell and Gahr, 2000; Nkrumah et al., 2006). This was illustrated in several studies where a clear segregation of rumen microbial profiles was seen in cattle with different RFI values. In particular, the ruminal bacterial profiles (but not the total numbers of bacterial cells) were different between steers divergent for RFI when fed both growing (Hernandez-Sanabria et al., 2010) and finishing diets (Guan et al., 2008; Hernandez-Sanabria et al., 2010). This segregation of ruminal microbial profiles was also seen between forage-fed beef LRFI and HRFI heifers (Carberry et al., 2012). Therefore, selection for LRFI animals favors a reduced carbon footprint, which appears to be mediated by intrinsic differences in the ruminal microflora of HRFI and LRFI animals.

#### RFI and its Relationship to Stress and Immune Response

Although several studies have been conducted to understand the relationship of RFI with important traits such as metabolism, fertility, and carcass characteristics, little is known about the contribution of the stress and innate immune responses to divergence in RFI in cattle. Munro et al. (2017) compared overnight heart rate as a measure of stress level between divergent RFI heifer calves, and found that LRFI heifer calves had a lower overnight heart rate when compared to their HRFI counterparts. Kelly et al. (2017) reported no difference in blood concentration of adrenocorticotropic hormone (ACTH) as well as neutrophil, lymphocytes, monocyte, eosinophils and basophil count, when divergent Simmental heifers were treated with standardized dose of ACTH (1.98 IU/kg metabolic BW^0.75^). ACTH is secreted from the anterior pituitary gland following a stressful event, which then stimulates the secretion of cortisol from the adrenal cortex (Colpoys, 2015). As well, a RFI × time interaction was seen for cortisol concentrations with HRFI displaying greater cortisol response than LRFI heifers from 40 to 150 min following ACTH administration. Consolo et al. (2018) reported greater lymphocyte counts with fewer segmented neutrophils in both LRFI pregnant and non-pregnant heifers compared to their HRFI counterparts. In addition, LRFI non-pregnant heifers showed higher levels of immunoglobulin M (IgM) than inefficient heifers. Results support potential synergistic association between improved feed efficiency and humoral defense as indicated by IgM concentration as well as positive association between improved feed efficiency and cellular defense as indicated by lymphocyte counts. Similarly, Herd et al. (2019) reported a negative correlation of RFI with white blood cells and lymphocyte counts in Angus cattle. Overall, the data suggest that LRFI animals may cope with physiological and pathogenic stress better than HRFI animals.

#### Relationships Between RFI and Fertility

Even though the reduction of feed costs is a major factor favoring the profitability of beef production, successful reproduction in cow-calf operations is also a key factor affecting profitability (Dickerson, 1970). A study published in 2012 reported that LRFI heifers reached puberty earlier, conceived at an earlier age, and delivered heavier calves compared to their HRFI counterparts (Smith, 2012). Contrary to this finding, several recent studies have found that LRFI heifers showed delayed onset of puberty (Shaffer et al., 2011; Randel and Welsh, 2013), but no difference was observed in pregnancy or conception rates between LRFI and HRFI heifers (Shaffer et al., 2011). When RFI was adjusted for body fatness (RFIfat), in an attempt to ensure independence with age at puberty in heifers, there was no difference in the percentage of LRFIfat and HRFIfat heifers reaching puberty (Schenkel et al., 2004; Basarab et al., 2007; Basarab et al., 2011). In terms of bull fertility, several studies have shown that scrotal circumference (SC) and semen characteristics were not statistically different between LRFI and HRFI bulls (Hafla et al., 2012; Wang et al., 2012). As well, a greater number of progeny per sire was seen for LRFI bulls in a multi-sire natural mating experiment (Wang et al., 2012). In contrast, other studies found that LRFI bulls had a smaller scrotal circumference (Johnson et al., 2019), decreased sperm motility (Wang et al., 2012; Awda et al., 2013; Johnson et al., 2019), decreased progressive sperm motility, increased abundance of tail abnormalities, and delayed sexual maturity (Fontoura et al., 2016; Montanholi et al., 2016). Moreover, when RFI was adjusted for backfat thickness, LRFIfat bulls displayed lower sperm motility, decreased progressive sperm motility, as well as a smaller scrotal circumference (Awda et al., 2013). These data suggest that some fertility issues may exist with certain LRFI animals.

In conclusion, the scientific community has reported that variation in RFI is associated with major physiological processes controlling feed intake and digestion, activity, body composition and metabolism, as well as thermoregulation. It has also been established that there are clear relationships between RFI and traits such as enteric methane production, immune response and fertility. Therefore, the differences in the way high and low RFI cattle use and partition energy might have differential effects upon the offspring of these animals when exposed to contrasting planes of prenatal nutrition. Going forward, it will be important to more clearly define the biological pathways underlying the biology of RFI in order to predict production responses in different environments that cattle can experience.

### Prenatal Nutrient Restriction

Maternal stress, particularly nutritional stress, is considered to be one of the major drivers of negative consequences arising during the developmental programming of offspring (Du et al., 2010 and 2011; Robinson et al., 2013; Caton et al., 2019). Research on children conceived prior to and during the Dutch Hunger Winter (between December 1944 and April 1945 in World War II) demonstrated that children exposed to poor nutritional conditions in early gestation had a decline in cognitive function, increased risk of coronary heart disease, and an atherogenic lipid profile, despite having a slightly higher than average birth weight (de Rooij et al., 2006; Roseboom et al., 2011). Similar to studies conducted on humans, maternal malnutrition during gestation can also affect postnatal growth and development, fertility, and the health of offspring in cattle (Long et al., 2009; Du et al., 2010; Funston et al., 2010; Du et al., 2011; Robinson et al., 2013; Caton et al., 2019). For the purposes of this discussion, maternal nutrient restriction includes any event which reduces nutrient supply to the fetus during critical developmental stages (Caton and Hess, 2010; Reynolds and Caton, 2012).

#### Effect of Timing of Prenatal Nutrient Restriction on Growth

The effects of maternal malnutrition on fetal development depends upon timing, level, and/or duration of the period of compromised nutrition (Reynolds and Caton, 2012; Reynolds et al., 2013; Vonnahme et al., 2015; Zhang et al., 2015). Nutrient insults in late gestation would be expected to have the greatest impact on fetal size (i.e., birth weight and body length), as almost 75% of ruminant fetal growth occurs at this stage (Du et al., 2010; Funston et al., 2010). Several studies have shown that nutrient insults during the last two-trimesters of a pregnancy can reduce fetal growth, birth weight, as well as number of muscle fibres and adipocytes in the offspring of sheep and cattle (Reed et al., 2007; Swanson et al., 2008; Du et al., 2010; Funston et al., 2010; Vonnahme et al., 2015; Long et al., 2021). Therefore, any decrease in birth and body weight because of nutrient insults at these later stages of pregnancy can lead to an impairment of adipogenesis and myogenesis as well as a decrease in muscle fibre growth (Du et al., 2010; Funston et al., 2010).

In beef cattle production, undernutrition during first half of gestation is considered to be of little postnatal significance since the fetus needs only a limited level of nutrition for growth and development at this stage compared to maternal needs (NRC, 1996; Robinson et al., 1999; NRC, 2007; NASEM, 2016). However, critical events such as placental development and organogenesis occur during early pregnancy. In terms of nutrient partitioning in the fetus during gestation, the priority is with essential organs (i.e., heart, liver, lung, brain, kidney, etc.) rather than skeletal muscle. Because no net increase in muscle fibre numbers occurs after birth, nutrient insults during early gestation may change nutrient partitioning to essential organs rather than skeletal muscle (Greenwood et al., 2000; Zhu et al., 2006; Long et al., 2009; Du et al., 2010; Funston et al., 2010; Du et al., 2011). Several studies have shown that calves born from dams that were nutritionally restricted during early-to mid-gestation and then re-alimented before parturition had similar ratios of weight gain to feed intake (Long et al., 2009; Long et al., 2012; Johnson et al., 2019). However, there is some evidence for increased weight gain between 10 and 16 months of age compared to their normally fed control groups (Johnson et al., 2019).

#### Effect of Prenatal Nutrient Restriction on Muscle Growth

As reviewed by Du and coworkers (2010), there are two stages of myogenesis in cattle development. During the first wave of myogenesis, which occurs within the first 2 months of gestation, the primary muscle fibres are formed (Du et al., 2010). Since only a limited number of muscle fibres are established at this stage, nutritional insults during this period likely have a negligible effect on fetal skeletal muscle development (Zhu et al., 2006; Du et al., 2010). However, the secondary and in fact majority of muscle fibers are formed during the second wave of myogenesis occurring between the 3rd and 8th months of gestation (Du et al., 2010), and therefore nutritional insults during this period appear to negatively affect fetal skeletal muscle development (Zhu et al., 2006; Du et al., 2010). It is also important to mention that skeletal muscle matures during late gestation, at approximately day 105 for ewes and day 210 for cattle (Du et al., 2010). Hence, nutrient restriction after this stage has no major influence on the number of muscle fibres, although it can impact their size (Greenwood et al., 1998). For example, McCoard et al. (2000) reported that nutrient restriction in late gestation did not affect the number of muscle fibres in lambs but it did reduce their diameter. Overall, the impact of prenatal nutrient restriction on muscle growth in cattle depends on its timing, with the majority of negative effects occurring between day 60 to day 210 of gestation, when the majority of myogenesis occurs.

#### Effect of Prenatal Nutrient Restriction on Carcass Quality

Several studies have shown that poor prenatal nutrition is not only associated with reduced growth and body weight of cattle, but also negatively impacts carcass quality (Greenwood et al., 2009; Long et al., 2012). Greenwood et al. (2009) reported lower birth weight, smaller carcasses, and smaller primal cuts in calves born to dams that were nutritionally restricted during gestation. Long et al. (2012) reported no significant differences in body weight or organ weights in calves born to dams that were nutritionally restricted from early-to mid-gestational undernutrition (70% of NRC recommendations) compared to a normally fed control group (100% of NRC recommendations). However, yield grade was reduced and semitendinosus weight/hot carcass weight (HCW) tended to be reduced in the diet-restricted group. In addition, average adipocyte diameter was increased in subcutaneous, mesenteric, and omental adipose tissues in the restricted group, and perirenal adipose tissue tended to be increased in restricted compared to non-restricted group. This suggests that maternal undernutrition in cattle may cause alterations in carcass quality of the offspring, potentially resulting in greater adiposity and reduced muscle mass. However, Blair et al. (2013) reported no differences in HCW, dressing percent, ribeye area, marbling score, and intramuscular fat between calves born from energy restricted vs. non-restricted cows during mid-gestation. As well, rib backfat and the USDA (the United States Department of Agriculture) yield grade tended to be lower in calves born from energy-restricted dams. Similarly, Mohrhauser et al. (2015) reported no influence on offspring HCW, dressing percent, LM area (LMA), marbling score, percent intramuscular fat, objective color, or Warner-Bratzler shear force (WBSF) between calves born from energy restricted vs. non-restricted cows during mid-gestation. They also reported greater ratio of marbling score to 12th rib fat thickness and ratio of percent intramuscular fat to 12th rib fat thickness in progeny from energy restricted cows. They suggested that maternal energy status during mid-gestation may impact fat deposition in intramuscular and subcutaneous fat depots without impacting muscle mass. These conflicting results show that more research is still needed to evaluate how maternal undernutrition may influence carcass traits in beef cattle offspring.

#### Effect of Prenatal Nutrient Restriction on Health

Several studies have been performed to understand the impacts of prenatal under- or over-nutrition on the health of cattle offspring. Mossa et al. (2013) reported enlarged aorta and increased arterial blood pressure in calves born to nutritionally restricted dams during first trimester of gestation compared with controls. Maternal nutrient restriction (∼75% of recommended allowance) during early gestation also compromised placental angiogenesis, cotyledon weight, and fetal development in beef cattle (Zhu et al., 2007). Corah et al. (1975) reported increased mortality rates and incidence of scours in calves born from dams having an energy-restricted diet (65% of their dietary energy requirements) in the last trimester of gestation compared to those born from non-energy-restricted dams (100% of their dietary requirements). From these studies it would appear that the health of calves exposed to prenatal nutritional restriction are visibly very negatively affected in aspects that are severely detrimental to productive and economic outcomes.

Passive immunity, *via* transfer of maternal antibodies to the fetus through placenta and to offspring through high quality colostrum, is an important factor in determining the health of offspring (Qi et al., 2012). A study by Hough et al. (1990) showed that colostral immunoglobulin G (IgG) concentrations were similar in both Angus cows that consumed just 57% of their needed nutritional requirements in the last trimester of pregnancy and non-feed-restricted cows, however, calves fed colostrum from diet-restricted dams tended to have lower IgG concentrations in serum. In contrast, Noya et al. (2019) reported that plasma concentrations of immunoglobulin M (IgM) and IgG were not different between calves born to dams nutritionally restricted during the first trimester of gestation compared to non-restricted calves. Stalker et al. (2006) reported no difference in concentration of serum IgG between 24 and 48 h after birth in calves born to dams receiving supplemental feed or not during the last trimester of gestation, while calf survivability from birth to weaning was greater in supplemented cows. Moriel et al. (2016) also reported similar serum IgG concentrations at birth and subsequent health and growth responses in Angus calves born to dams exposed to energy-restricted diet (70% of their energy requirements) during the last 40 days of gestation compared to that of non-restricted ones (100% of their energy requirements). All calves from both groups were vaccinated against BRD pathogens 10 days after weaning, and calves from energy-restricted cows had reduced serum concentrations of cortisol, haptoglobin, and antibody titers against bovine viral diarrhea virus-1 upon vaccination. They concluded that prenatal undernutrition, even if short term, impaired vaccination-induced physiologic responses required for proper immunity against BRD pathogens. Therefore, it appears there is quite strong evidence that prenatal maternal nutrition is an important factor in determining the immunological health of progeny animals.

#### Effect of Prenatal Nutrient Restriction on Fertility

In terms of the effect of maternal nutrient restriction on the fertility of ruminant offspring, a number of effects have been observed. These include a decreased ovulation rate (Rae et al., 2002a), decreased ovarian weight (Long et al., 2012), and a reduced number of postnatal follicles (Mossa et al., 2013) in female progeny. These effects were observed when dams were nutritionally restricted during early-to mid-gestation and then re-alimented thereafter. In both sheep and cattle, there was no significant effect on reproductive development and adult reproductive function in rams or bull progeny, when ewes or heifers were nutritionally restricted during early-to mid-gestation and re-alimented thereafter (Rae et al., 2002a; Rae et al., 2002b; Johnson et al., 2019). However, delayed prostate development has been observed in male rat pup progeny, when Wistar rat dams were fed a protein-restricted diet throughout the entire gestational period (Rinaldi et al., 2013). In addition, nutrient insults during pregnancy resulted in male lamb progeny with delayed puberty, decreased plasma testosterone concentrations (from birth until 28 weeks of age) and decreased testicular volume (from birth until 35 weeks of age) (Da Silva et al., 2001). Therefore, maternal undernutrition can affect reproductive development and function in both male and female progeny, although the long-term effects (beyond 35 weeks of age) have not been investigated.

In summary, the availability of nutrients to the dam and the fetus at different stages of gestation can affect the growth and development in the progeny animals. Important production and economic traits in beef cattle such as muscle growth, carcass quality, health, as well as reproductive development and function can be affected. These findings depict the main drawbacks of prenatal undernutrition at different stages of gestation in cattle and highlight that a more thorough understanding of how prenatal nutrition affects postnatal growth and development, and by which mechanisms it does this, is vital to optimize production efficiencies.

### Molecular Characterization of RFI in Cattle Experiencing Prenatal Malnutrition Stimuli

One important aspect of the RFI phenotype is that it is typically measured in a feedlot setting under *ad libitum* intake. The main cattle herd that produces feedlot animals generally does not exist in an environment of unlimited feed resources, is pregnant for approximately 77–78% of the year, and is feeding a calf for about 50% of the year. As selection for RFI has inherent effects on the ways animals acquire, metabolize, and distribute energy, it is important to investigate how selection for RFI might affect gestating cattle under various environmental conditions. This is especially important as the nutritional environment the fetus is exposed to can have long lasting effects on postnatal growth and development, as reviewed above.

There appears to be evidence that supports a differential response between high and low RFI cattle to different planes of prenatal nutrition, as well as an absence of response. Johnson et al. (2019) studied the impact of genetic potential for RFI and prenatal undernutrition during early-to mid-gestation in beef heifers on the growth and reproductive potential of progeny bulls. They reported that LRFI progeny bulls reached puberty at a later age, and had smaller scrotal circumferences regardless of prenatal diet fed. They also found that bulls exposed to the restricted prenatal diet were heavier between 10 and 16 months of age. Therefore, no interaction between prenatal diet and genetic potential for RFI upon offspring performance metrics measured was detected. Meale et al. (2021) reported on a similar experimental design as Johnson, but the male progeny had been castrated at birth. They reported a trend for heavier final slaughter weights, but significantly lower dressing percentage in steers from HRFI parents where the dam was exposed to a Low-diet (75% of recommended feed intake) during gestation compared to that of HRFI/Moderate-diet (100% of recommended feed intake), LRFI/Low-diet, and LRFI/Moderate-diet. Therefore, HRFI/Low-diet steers appeared heavier at slaughter, but actually did not produce a heavier carcass. The difference in results between the two studies could be the result of the sex differences between the offspring, or sample sizes, but they also demonstrate a possibility of differential response between low and high RFI cattle and/or their offspring, to prenatal diet, which can be exploited to understand how selection for RFI will affect the mature cowherd.

The application of high-throughput omics technologies has revolutionized biological research by allowing scientists to explore the molecular details of complex biological systems. These high-throughput omics techniques employ genomics, epigenomics, transcriptomics, proteomics, and metabolomics. Bringing together multiple omics approaches to look at a biological problem offers researchers the opportunity to explore the system, at a molecular level, from many different perspectives. These technologies also allow researchers to investigate the effects of environmental perturbations on underlying biological pathways related to important production traits in livestock, even if there are no clear visual physiological effects due to the timing or subtlety of their expression. This section describes and discusses the application of three different omics sciences including metabolomics, transcriptomics and epigenomics, to explore the biological pathways underlying RFI, prenatal diet, or their interaction in beef cattle. By creating an external stimuli that places animals with different genetic potentials for RFI in different environments, we can more thoroughly tease out biological pathways associated with RFI by contrasting their molecular relationships relative to those environments.

#### Metabolomic Signatures of RFI, Prenatal Nutrition, and Their Interaction

Metabolites are considered the end products of cellular regulatory processes occurring inside the cell (many of which are guided by the genome) as well as events, exposures or phenomena occurring outside the cell or organism, which are dictated by the environment (Fiehn, 2002; Goldansaz et al., 2017). As a result, measuring the metabolome, which is defined as the complete set of metabolites in a cell, tissue, organ, or organism, can help to reveal key interactions between genes and the environment. In other words, metabolomics allows researchers to gain a more complete understanding of an organism’s chemical phenotype (Bouatra et al., 2013; Monteiro et al., 2013).

Metabolomics uses advanced analytical chemistry techniques to comprehensively detect and measure hundreds of small molecule metabolites in cells, biofluids, and tissues. Metabolomics uses a variety of analytical chemistry techniques including nuclear magnetic resonance (NMR) spectroscopy and mass spectrometry (MS) as well as coupled techniques such as liquid chromatography-mass spectrometry (LC-MS), gas chromatography-mass spectrometry (GC-MS), and inductively coupled plasma-mass spectrometry (ICP-MS). Each technique enables the detection of specific classes of metabolites with differing levels of sensitivity and accuracy. Each technique has also been used to identify and quantify different metabolites within various bovine biofluids and tissues and all of these metabolites are available in public databases (Foroutan et al., 2019a; Foroutan et al., 2019b; Foroutan et al., 2020b; Foroutan and Wishart, 2021).

Metabolomics has been used to predict RFI (Kelly et al., 2010; Fitzsimons et al., 2013; Karisa et al., 2014; Foroutan et al., 2020a), and to explore the molecular physiological or biochemical underpinnings of lean tissue or muscle content in livestock. For example, Lawrence et al. (2011) reported a positive correlation between muscularity and concentration of creatinine in blood. Creatinine is produced *via* creatinine phosphate and typically is increased due to muscle breakdown, and therefore is an indicator biological pathways related to protein metabolism. In sheep, blood creatinine concentrations have been shown to have a negative correlation with fat depth and a positive correlation with muscle mass (Cameron, 1992; Clarke et al., 1996). As LRFI cattle have shown a propensity for traits associated with leanness in several studies (Arthur et al., 1997; Arthur et al., 2001; Richardson et al., 2001), observing differences in circulating creatinine might be plausible for populations of cattle where an association between RFI and body leanness, muscularity, and/or carcass fatness occurs. In fact, lower concentrations of creatinine have been found in the plasma of HRFI vs. LRFI beef cattle (Fitzsimons et al., 2013), although Karisa et al. (2014) found higher levels of creatine, the precursor to creatinine, in the blood of HRFI cattle. Another circulating metabolite associated with protein metabolism, urea, has also been seen to have a negative correlation with lean growth of sheep (Cameron, 1992; Clarke et al., 1996), and in the blood of weaned Angus steers, urea concentrations tended to have a positive association with RFI (Richardson et al., 2004; Fitzsimons et al., 2013). Understanding the biological pathways associated with RFI in the area of protein metabolism, may also help negate the selection of unwanted correlations between RFI and carcass lean or fat levels.

Other metabolites associated with energy and lipid metabolism such as glucose (associated with insulin resistance and energy metabolism), carnitine (associated with glucose, lipid and energy metabolism), and beta-hydroxybutyrate (product of tissue fatty acid catabolism) have been found to be higher in the plasma of HRFI vs. LRFI beef cattle (Kelly et al., 2010; Fitzsimons et al., 2013; Karisa et al., 2014). Recently, Foroutan and coworkers used three metabolomics platforms including NMR, ICP-MS, and LC-MS/MS to compare a wide range of metabolites in the serum of young, divergent RFI Angus bulls (Foroutan et al., 2020a). They found two sets of predictive RFI biomarkers; one included formate (associated with energy metabolism and immune response) and leucine (associated with protein and energy metabolism as well as immune response) (best for NMR), and another set included butyrylcarnitine (associated with energy metabolism and immune response) and LysoPC28:0 (associated with lipid metabolism and immune response) (best for LC-MS/MS), both with high sensitivity and specificity (Area Under the Receiver Operating Characteristic Curve or AUROC >0.85), for distinguishing HRFI from LRFI animals. Taken altogether, there is clear evidence that a number of specific blood metabolites differ between LRFI and HRFI animals, especially those associated with protein, energy, and lipid metabolism, as well as immune response. These data suggest that metabolomics offers a good opportunity to subset molecular pathways that would help detect additional markers of RFI propensity and augment genomic prediction.

While a critical mass of metabolomic data has been collected and published on RFI, there is very little published, large-scale metabolomic data on prenatal diet restriction and their effects on progeny. Indeed, a literature review conducted by us revealed only a few studies that investigated metabolite levels or metabolite changes in the progeny of livestock or other animals that were fed restricted or enhanced diets. For example, Zambrano et al. (2006) reported that male offspring of rats exposed to a protein-restricted diet during pregnancy showed evidence of insulin resistance *via* a glucose tolerance test, and had higher levels of resting blood cholesterol and triglycerides than the non-restricted group. Ford et al. (2007) reported that male offspring of ewes experiencing nutrient restriction during early-to mid-gestation and re-alimented afterwards had higher blood glucose levels than those of a control group. Considering glucose is also a candidate metabolite to be influenced by RFI, it would be beneficial to see if cattle divergent for RFI would respond differently in terms of blood glucose levels, if they were subjected to different prenatal nutritional treatments. This comparison, in concert with observations of corresponding phenotypic responses, would shed some light on the biological pathways underlying both conditions and contribute to explaining variability in profiles between different metabolite studies of divergent RFI cattle. Metabolomics studies are an important step to help identify key metabolites and biological/physiological pathways that can play a part in the manifestation of effects of prenatal nutrition on post-natal growth and development.

#### Transcriptomic Signatures of RFI, Prenatal Nutrition, and Their Interaction

While metabolomics allows one to explore downstream changes associated with physiological perturbations or phenotypic differences, transcriptomics is the next upstream step controlling metabolic and phenotypic differences that is amendable to large-scale quantification. Transcriptomics has been widely used in livestock studies (Kelly et al., 2011; Al-Husseini et al., 2014; Paradis et al., 2015), and utilizes advanced molecular biology techniques to comprehensively measure expression of dozens to thousands of transcripts in cells, biofluids and tissues. Such techniques include microarrays or gene-chips, RNA-seq, and quantitative polymerase chain reaction (qPCR).

Studies examining gene expression in relation to RFI are extensive, and the biological information they have generated has moved past the discovery stage. For example, expression values of selected genes are now being assessed to predict RFI phenotype. Al-Husseini et al. (2014) tested genes expressed in liver that were discovered by Chen et al. (2011, 2012) to be associated with RFI. They used regression and prediction equations to determine the variance in RFI explained by gene expression, and its predictive ability of RFI, respectively. Genes associated with lipid metabolism, cellular growth and proliferation (alpha-2-HS-glycoprotein [*AHSG*], growth hormone receptor [*GHR*] and inhibin, beta A [*INHBA*]), were negatively correlated with RFI, and genes associated with fibrinolysis, coagulation, and development and regulation of cell differentiation (protocadherin 19 [*PCDH19*], S100 calcium-binding protein A10 [*S100A10*], serpin peptidase inhibitor, clade I (pancpin), member2 [*SERPINI2*], superoxide dismutase 3, extracellular [*SOD3*], and glutathione S-transferase mu 1 [*GSTM1*]), were positively associated with RFI. Although all genes explained variation in RFI phenotype, different subsets of these genes explained more variation for high or low RFI groups, identifying the fact that genes may act differently at the ends of the RFI spectrum seen in cattle populations. Altogether, selective gene expression explained 32.2% of the variance in RFI across all animals in the study, and explained 76.3 and 79.5% of the variation in RFI in low and high RFI groups, respectively.

A significant proportion in the variance of RFI is predicted to involve pathways important to mitochondrial function (Bottje and Carstens 2009; Herd and Arthur 2009). Kelly et al. (2011) investigated differential expression of genes associated with cellular energetic efficiency in the *Longissimus dorsi* muscle of LRFI compared to HRFI cattle, including peroxisome proliferator-activated receptor gamma coactivator 1-alpha (PGC-1α) [*PPARGC1A*], upregulated in HRFI cattle, and uncoupling protein 3 [*UCP3*], upregulated in LRFI cattle. They also utilized a diet (high forage versus low forage) by RFI (high versus low) interaction to identify expression differences in ADP/ATP translocase T1 [*ANT1*] and cyto c oxidase II [*COX II*]. Both the Al-Husseini et al. (2014) and Kelly et al. (2011) studies emphasize the utility of using transcriptomics to predict RFI, and how biological pathways related to RFI can change over the RFI spectrum, and in different environments. These aspects of investigation will ultimately bring more robustness to the use of gene expression as an aid in genomic prediction of RFI, especially across different populations and geographical locations.

In addition to the genes and pathways identified above, a number of physiological differences with regard to immune and stress response are often detected between HRFI and LRFI animals. Genes associated with inflammatory processes, including hemoglobin β [*HBB*], myxovirus resistance one interferon-inducible protein p78 [*MX1*], ISG15 ubiquitin-like modifier [*ISG15*], hect domain and RLD 6 [*HERC6*], and interferon-induced protein 44 [*IFI44*] have shown differential expression patterns in the liver between HRFI and LRFI heifers (Paradis et al., 2015). Another gene involved with activation of stress response, but also with activation of muscle-specific, growth factor-induced genes, myocyte enhancer factor 2A [*MEF2A*], was reported to have higher expression in *Longissimus thoracis* and *semimembranosus* muscles, liver, and testis of LRFI Angus bull offspring compared to their HRFI counterparts (Foroutan et al., 2021). *MEF2A* binding has also been identified upstream of muscle creatine kinase (Molkentin and Markham, 1993), which possibly links differences in *MEF2A* expression with differences in circulating creatine or creatinine as discussed in the previous section, and the occasional differences detected in lean muscle accretion in divergent RFI cattle. Expression of the *MEF2A* gene may also be related to serum metabolites of RFI including those associated with muscle protein synthesis (leucine, serine, glycine, Ca2+, and proinsulin) and oxidative stress (formic acid, LysoPC(28:0), L-carnitine, butyrylcarnitine, and propionyl-L-carnitine), in agreement with the results of studies where LRFI cattle showed potential differences in immune response and stress, and whole body chemical protein level compared to their HRFI counterparts (Foroutan, 2021).

As mentioned earlier, it is important to ensure that selection for RFI in cattle does not negatively affect their reproductive capacity. Johnson et al. (2020) identified 17 differentially expressed genes involved in molecular networks associated with reproduction and fertility in testis of divergent RFI Angus bulls. Of particular interest were insulin like growth factor 1 receptor [*IGF1R*], phospholipase C delta 1 [*PLCD1*], and inositol polyphosphate-4-phosphatase type II B [*INPP4B*], where higher expression of *IGF1R* and lower expression of *PLCD1* and *INPP4B* could activate PI3K–Akt signaling responsible for cell growth, proliferation and steroid metabolism in HRFI bulls. Studies such as this one could contribute to identifying genes and pathways that if selected along with RFI, or are a part of genetic variation contributing to the RFI phenotype, would be undesirable as they may also negatively impact fertility. The goal of their use in genomically enhanced selection in cattle would be to ensure fertility is not negatively affected at the genetic level by selection for RFI.

As compared to RFI, similar efforts have been put towards using transcriptomics to investigate prenatal nutritional effects on gene expression in the offspring. Several genes involved in related pathways to those expected to play a part in determining RFI, have been identified as affected by prenatal nutrition. Members of the insulin like growth factor family (insulin like growth factor 1 [*IGF1*], its receptor *IGF1R,* insulin like growth factor 2 receptor [*IGF2R*]), as well as insulin receptor [*INSR*], have been identified in at least one or several studies examining effects of prenatal nutrition in cattle (Micke et al., 2011; Wang et al., 2015; Paradis et al., 2017). Myogenesis related genes, myogenic differentiation 1 [*MYOD1*], myogenin [*MYOG*], and *MEF2A* have also been identified as differentially expressed due to prenatal diet (Paradis et al., 2017; Foroutan et al., 2021), as well as leptin [*LEP*] (associated with energy homeostasis), peroxisome proliferator activated receptor gamma [*PPARG*] (associated with adipocyte differentiation), and protocadherin 19 [*PCDH19*] (associated with immune function) (Micke et al., 2011; Paradis et al., 2017). Diniz et al. (2021) utilized multi-tissue transcriptomics to explore the regulatory relationships between genes in fetal cerebrum, liver, and muscle tissues to shed light on the putative mechanisms that underlie the effects of early maternal nutrient restriction on bovine developmental programming. By detecting genes that were differentially co-expressed and differentially connected, and identifying transcription factors and gene hubs modulating the expression of differentially expressed genes, the authors identified regulators of energy homeostasis, inflammatory responses, cellular stress, myogenesis, and nutrient-sensing signaling pathways, whose regulation have been previously identified as being affected by prenatal nutrition.

To date only two published articles have evaluated the impact of the interaction between both prenatal diet and RFI on gene expression. Johnson et al. (2020) used RNA-seq to identify differentially expressed genes in testis of bull offspring born from divergent RFI parents, of which the pregnant females were also exposed to differential planes of prenatal nutrition during the first half of gestation. These authors did not detect any genes that were significantly affected by both parental RFI and prenatal nutrition in testis. Foroutan et al. (2021) observed an interaction between parental RFI and prenatal diet in *Longissimus thoracis* muscle; mRNA abundance of *MEF2A* was higher in moderate prenatal diet-LRFI bulls compared to other three groups (moderate diet-HRFI, low diet-LRFI, and low diet-HRFI). With these two publications we start to get a glimpse on how cattle selected for divergent RFI react, or can be stoic to, environmental stressors. For example, LRFI bulls might be predisposed to responding to the differential prenatal diet in muscle tissue, but not in reproductive tissue. Alternatively, the timing of the prenatal nutritional insult may have had a greater effect upon muscle growth than testis development, and LRFI cattle might be poised to take greater advantage of more abundant prenatal nutritional resources than HRFI cattle.

#### Epigenomic Signatures of RFI, Prenatal Nutrition, and Their Interaction

Epigenomics offers a layer of genomic regulation that can link environmental influences to transcriptomic changes, and ultimately differences in metabolites and phenotype. Epigenetics is defined as the study of heritable changes in gene expression, resulting from alterations in chromatin structure but not alterations in the DNA sequence (Funston and Summers, 2013; Romanoski et al., 2015). The epigenome is represented by chemical changes to DNA and DNA-associated proteins including histone modifications, chromatin remodeling and DNA methylation, as well as non-coding RNAs, all of which alter mechanisms of gene expression. Once epigenomic compounds attach to or are removed from a given DNA strand, or attach to specific histones and modify their function, they can change the way cells use instructions encoded in DNA. In this manner, epigenetic processes control the timing and intensity of gene expression (Zeisel, 2009; McKay and Mathers, 2011). Epigenetic modifications can be passed from one generation to the next if the DNA of gametes are affected, or from cell to cell as cells divide. An organism’s epigenome is dynamic throughout its lifetime, and can be altered as a result of changes in lifestyle and environmental factors (i.e., diet, stress, toxins, particulate air pollutant matter and infectious disease), which can expose an organism to pressures that prompt the appearance or alteration of epigenomic marks (Keil and Lein, 2016; Martin and Fry, 2018). However, the ability of the epigenome to adjust to these life pressures appears to be required for normal health and development in most organisms (Kanherkar et al., 2014).

As RFI is moderately heritable (Arthur et al., 2001; Basarab et al., 2003; Nkrumah et al., 2007), examining the epigenome for regulation of RFI at the molecular level may not have received a lot of attention. Now as epigenomic data has started to accumulate, it has shown its usefulness in terms of explaining genetic variance and contributing to phenotypic trait heritability (Xiang et al., 2019), and contributing to enrichment of GWAS analysis (Fang et al., 2020). A few recent studies have specifically examined DNA methylation patterns in reference to RFI phenotypes. Rocha et al. (2019) identified 1,493 differentially methylated cytosines (DMCs) and 279 differentially methylated regions (DMRs) in the hepatic tissue of Nelore cattle exhibiting extremes in RFI but unfortunately, they did not identify relationships between these DMCs/DMRs and specific biological pathway(s). Going a step further into the biology of RFI, Foroutan (2021) compared methylation in sperm DNA between HRFI and LRFI bulls and identified 1,400 DMRs, where HRFI sperm displayed 57% of the hypermethylated DMRs. The DMRs were associated with four networks including “embryonic development,” “DNA replication, DNA repair, and RNA processing,” “growth control and homeostasis,” as well as “lipid metabolism,” the latter two networks echoing a couple of the major physiological processes associated with RFI, i.e., body composition and metabolism. In a targeted analysis, Devos et al. (2021) measured DNA methylation in important regulators of growth and development including *IGF2* DMR2, *IGF2*/*H19* ICR (intergenic control region), and *IGF2R* DMR2, between parental LRFI and HRFI heifer and steer calves. All three DMRs were hyper-methylated in the liver of LRFI steers. As well, *IGF2*/*H19* ICR was hyper-methylated in the *Longissimus dorsi* muscle of LRFI steers at birth, providing some evidence that consistent hyper-methylation of these DMRs may underlie some variation in RFI.

Maternal nutrient status can cause epigenetic modifications to the genome of the developing fetus, which potentially can impact growth, feed efficiency, fertility and health of future generations of cattle (Funston and Summers, 2013). Expression of genes associated with epigenetic modifications to DNA, for example those involved with methylation, phosphorylation, acetylation, de-acetylation, and ubiquitination, have been found to be altered by prenatal nutrition in bovine fetal liver (Crouse et al., 2017). Imprinted genes have also been commonly investigated for effects of prenatal nutrition upon the epigenome, as their mono-allelic expression is commonly under the influence of DNA methylation, and after one of the first published differences in DNA methylation due to prenatal diet in humans was for the imprinted gene *IGF2* (Heijmans et al., 2008). Altered methylation levels corresponding to prenatal diet for CpG islands of both *H19* and *IGF2R*, as well as *IGF2* DMR2 and *IGF2R* DMR2, have been detected in sheep offspring (Lan et al., 2013), and cattle (Paradis et al., 2017; Devos et al., 2021), respectively. A comparison between restricted and moderate prenatal diet effects upon methylation levels in bull offspring identified 652 DMRs in their sperm DNA, of which 77.8% were hyper-methylated in the restricted group. The DMRs were associated with three networks including “cell survival and growth,” “disease or abnormalities,” and “connective tissue development” and many of the genes in these networks are important for normal spermatogenesis and male fertility (Foroutan et al., 2021). Devos et al. (2021) also investigated the interaction between parental genetic potential for RFI and prenatal diet upon DNA methylation. In steer offspring, they found significant alterations in methylation level of *IGF2* DMR2, *IGF2*/*H19* ICR, and *IGF2R* DMR2 in *Longissimus dorsi* muscle, where at birth steers exposed to the moderate prenatal diet, and belonged to the LRFI group, displayed higher levels of methylation of many individual CpGs within each DMR. In heifer calves, at birth, they also observed that animals exposed to the moderate prenatal diet, and belonged to the LRFI group, displayed higher levels of methylation in *IGF2* DMR2 of *Longissimus dorsi* muscle, although methylation patterns for the other DMRs were not similar to the steers. These data suggest that genetic potential for RFI in combination with prenatal diet, induces tissue- and sex-specific alterations in the DNA methylation pattern of beef cattle, and may alter some similar pathways, especially under the broad themes of growth and development within skeletal muscle. These results highlight the need for more encompassing research on DNA methylation differences between tissues, sexes, and throughout the lifetime of the postnatal animal, as these factors will affect how the data is incorporated into any type of genetic merit indices or estimations of phenotypic performance.

#### Integration of Multiple Omics Sciences in the Beef Industry

Integration of multiple “omics” studies to improve predictions of the genetic merit of livestock and to understand biological pathways are well underway or on the immediate horizon. Genome-wide association studies (GWAS) in cattle have been performed to identify metabolomic and several classes of transcriptomic QTLs (expression, splicing, allele-specific QTLs), and epigenetic information such as non-coding RNA QTLs, while genome wide methylation and histone modification data is becoming more readily available. Omic and other functional genetic information are contributing to the understanding of the genetic architecture of a phenotypic trait through its use to subset DNA variants that are more likely to be related to causal variants of trait variability. These subsets can then be used in GWAS and genomic prediction to explain greater amounts of heritability and have greater efficacy of use across populations (Snelling et al., 2013; Xiang et al., 2019; Fang et al., 2020; Ariel et al., 2021). RNA-Seq data has also been used to impute to the genomic DNA sequence level and used in conjunction with a GWAS analysis *via* a transcriptome-wide association study (TWAS) to identify gene-tissue pairs associated with production traits (Liu et al., 2020). This latter study also gave rise to The cattle Genotype-Tissue Expression atlas (https://cgtex.roslin.ed.ac.uk/). cGTEx was developed to help facilitate the functional understanding of genetic variants in the cattle genome. Considerable efforts are also being placed on the systematic measurement and organization of gene expression and other functional information such as single-cell atlases, areas of conserved genomic sequence across species, and the development of tools to test gene and genetic variant functionality, by the FAANG Consortium (Clark et al., 2020). These efforts, and ultimately linkages between functional information and genomic variation, will accelerate genetic selection in cattle breeding programs.

## Conclusion

Maximizing the efficiency and growth potential of beef cattle requires not only genetic selection (i.e., selection for feed efficiency, RFI or ADG) but also adequate nutrition throughout all stages of gestation in the developing fetuses. Although both RFI and maternal nutrition during gestation can affect progeny traits, our understanding of the biological mechanisms by which this occurs is still at a broad physiological level. Exploring the interaction between the genetic potential for RFI and an environmental stimulus such as prenatal nutrition will help the scientific community determine how selection for RFI affects the physiology of the gestating cow and her offspring, and how they both may be more or less resistant to environmental influences. An improved understanding of the influences of RFI, prenatal nutrition and their interaction, on progeny traits can be achieved with the help of multiple omics technologies, especially if differences in gross physical phenotypes are subtle, or are manifested later in life. Gene and pathway information generated by exposing high or low RFI cattle to different environments will also help identify those pathways or phenotypes that may be co-selected with low RFI, and that may negatively affect other production traits. Integration of data generated by omics technologies shows a great deal of promise to help animal scientists understand the molecular and biological regulatory mechanisms underlying any important physiological trait in cattle, and will contribute to the improvement of genetic merit cattle and the efficiency by which animal breeders can perform genetic selection.
